# Phenazine-Producing Rhizobacteria Promote Plant Growth and Reduce Redox and Osmotic Stress in Wheat Seedlings Under Saline Conditions

**DOI:** 10.3389/fpls.2020.575314

**Published:** 2020-09-29

**Authors:** Peiguo Yuan, Huiqiao Pan, Emily N. Boak, Leland S. Pierson, Elizabeth A. Pierson

**Affiliations:** ^1^Department of Horticultural Sciences, Texas A&M University, College Station, TX, United States; ^2^Molecular and Environmental Plant Sciences Program, Texas A&M University, College Station, TX, United States; ^3^Department of Plant Pathology and Microbiology, Texas A&M University, College Station, TX, United States

**Keywords:** salt stress, plant growth promoting rhizobacteria, wheat, phenazine, reactive oxygen species, plant-microbe interaction

## Abstract

Application of plant growth promoting bacteria may induce plant salt stress tolerance, however the underpinning microbial and plant mechanisms remain poorly understood. In the present study, the specific role of phenazine production by rhizosphere-colonizing *Pseudomonas* in mediating the inhibitory effects of salinity on wheat seed germination and seedling growth in four different varieties was investigated using *Pseudomonas chlororaphis* 30-84 (wild type) and isogenic derivatives deficient or enhanced in phenazine production. The results showed that varieties differed in how they responded to the salt stress treatment and the benefits derived from colonization by *P. chlororaphis* 30-84. In all varieties, the salt stress treatment significantly reduced seed germination, and in seedlings, reduced relative water content, increased reactive oxygen species (ROS) levels in leaves, and in three of four varieties, reduced shoot and root production compared to the no salt stress treatment. Inoculation of seeds with *Pseudomonas chlororaphis* 30-84 wild type or derivatives promoted salt-stress tolerance in seedlings of the four commercial winter wheat varieties tested, but the salt-stress tolerance phenotype was not entirely due to phenazine production. For example, all *P. chlororaphis* derivatives (including the phenazine-producing mutant) significantly improved relative water content in two varieties, Iba and CV 1, for which the salt stress treatment had a large impact. Importantly, all *P. chlororaphis* derivatives enabled the salt inhibited wheat varieties studied to maintain above ground productivity in saline conditions. However, only phenazine-producing derivatives enhanced the shoot or root growth of seedlings of all varieties under nonsaline conditions. Notably, ROS accumulation was reduced, and antioxidant enzyme (catalase) activity enhanced in the leaves of seedlings grown in saline conditions that were seed-treated with phenazine-producing *P. chlororaphis* derivatives as compared to noninoculated seedlings. The results demonstrate the capacity of *P. chlororaphis* to improve salt tolerance in wheat seedlings by promoting plant growth and reducing osmotic stress and a role for bacterial phenazine production in reducing redox stress.

## Introduction

Increasing soil salinity is a worldwide problem that is detrimental to plant growth, crop production, food security, and the livelihoods of farmers. Recent global estimates suggest that soil salinity affects almost one billion ha of arable land (Shrivastava and Kumar, 2015; Shahid et al., 2018) and causes billions of dollars in crop yield losses annually (Qadir et al., 2014; Abiala et al., 2018). Saline soils are defined as having an electrical conductivity of the saturation extract (ECe) greater than 4 dS/m (approximately 40 mM NaCl) at 25°C (Richards, 1954), and at this ECe, yields of most crops are reduced, although many crops exhibit yield reductions at lower values (Munns, 2005). Under severe salinity (ECe > 16dS/m, ~160 mM NaCl), average yields of most agronomically important crops such as rice, wheat, maize, sorghum, sugarcane, potato, sugar beet soybean and sweet potato, are reduced by 50% (Panta et al., 2014). For instance, wheat stressed at 100–175 mM NaCl showed delayed floral initiation and significant reduction in spikelet number, resulting in poor grain yields (Munns and Rawson, 1999). In addition to ion type and salt concentration, soil type, plant species (or varieties), plant developmental stage, stress distribution, cultivation system and weather conditions are strong determinants of the impacts of salt stress on plants (Morton et al., 2019).

Salinity impairs plant growth and development *via* a combination of factors including osmotic stress, nutrient deficiency, ion toxicity, and oxidative stress (Farooq et al., 2015; Isayenkov and Maathuis, 2019). Due to the high osmotic potential of saline soil water, plants are less efficient in absorbing water and nutrients, but may have excessive uptake of sodium (Na^+^) and chloride (Cl^−^) ions. Due to ion competition, plant deficiencies of several nutrients (Ca^2+^, K^+^, Mg^2+^, and NO_3_^−^) and nutritional imbalances may occur. Salt stress also causes an imbalance in the production of reactive oxygen species (ROS) and antioxidant defense compounds so accumulation of ROS is typical. These factors lead to poor germination, closure of stomata, inhibition of leaf cell expansion, reduced rates of transpiration, photosynthesis, and other metabolic processes, retarded plant growth and development, leaf senescence, reduction in productivity, and ultimately loss of yield (Yuan et al., 2018a; Morton et al., 2019; Zelm et al., 2020).

Plants have evolved diverse mechanisms to tolerate salinity but primarily do so *via* ion avoidance and exclusion and osmotic and tissue tolerance (Farooq et al., 2015; Reddy et al., 2017). Ion exclusion occurs in roots *via* mechanisms affecting ion uptake and transport but includes Na^+^ export from the xylem back into the soil. Salt stress avoidance *via* ion exclusion may be improved by the production of barriers to ion uptake, including suberization of the exo- and endodermis and increasing root cap development and border cell and mucilage production (Munns and Tester, 2008; Hawes et al., 2016). Osmotic salinity tolerance occurs when the plant can maintain water use efficiency *via* regulation of stomatal conductance and leaf expansion, similar to drought stress tolerance. Osmotic tolerance is regulated by phytohormones and long-distance signaling mechanisms (Chinnusamy et al., 2006). For example, salt-stressed plants may synthesize phytohormones such as abscisic acid (ABA) leading to stomatal closure to promote water use efficiency (Younis et al., 2003; Zhu, 2016). Tissue tolerance involves sequestration of Na^+^ in vacuoles, synthesis of compatible solutes, and production of enzymes catalyzing detoxification of ROS. Plants may accumulate compatible solutes such as proline, soluble sugars, glycine betaine, organic acids, trehalose and other osmolytes, to alleviate the osmotic stress at the cellular level (Liang et al., 2018). They may upregulate ROS scavenging enzymes such as superoxide dismutase, peroxidase and catalase to mitigate secondary oxidative stress under salt conditions (Yuan et al., 2018b; Zelm et al., 2020). The relative importance of stress tolerance mechanisms varies among crops and varieties, and knowledge of salt tolerance mechanisms is essential for the development and management of salt tolerant crops (Morton et al., 2019; Zelm et al., 2020).

Increasingly the use of plant-associated microorganisms, especially rhizosphere-colonizing and endophytic bacteria and fungi, is being studied as a rapid and cost-effective strategy to augment the development of stress tolerant crop varieties (Nadeem et al., 2014; Shrivastava and Kumar, 2015; Numan et al., 2018). It is well established that phytobiome members, especially rhizosphere and root dwellers, can play important roles in mediating abiotic stresses tolerance *via* promotion of plant growth, enhancement of nutrient availability, disease control, and modulation of plant abiotic stress signaling and response pathways (Yang et al., 2009; Hardoim et al., 2015; Reinhold-Hurek et al., 2015; de Vries et al., 2020). With regard to salinity, previous studies focused on plant growth-promoting rhizobacteria (PGPR) showed that they may alleviate salinity induced osmotic stress, nutrient deficiency, oxidative stress, and ion toxicity *via* the production of bioactive compounds and modifications to the rhizosphere environment. For example, PGPR were shown to alleviate salt stress *via* the production or metabolism of phytohormones triggering osmotic responses; the production of siderophores, phosphate solubilizing compounds, and other traits important for improved nutritional status; the production of antioxidants involved in ROS degradation and management of redox stress; and the production of an exopolysaccharide (EPS) matrix, which improves soil structure, increases soil water retention, and reduces available Na^+^ (Shrivastava and Kumar, 2015; Numan et al., 2018; Xiong et al., 2019).

Previous studies showed that phenazine-producing pseudomonads have diverse growth promoting capabilities. Phenazines are a class of diffusible, heterocyclic compounds each having substitution of various functional groups on the core phenazine ring structure (Mavrodi et al., 2001; Mavrodi et al., 2006; Biessy and Filion, 2018). Phenazines were shown to inhibit a diversity of plant pathogens and suppress the plant diseases they cause (Thomashow and Weller, 1988; Chin-A-Woeng et al., 2001; Pierson and Pierson, 2010; Cezairliyan et al., 2013; Zhou et al., 2016; Yu et al., 2018b). Phenazine production is important for the rhizosphere competence of producers (Mazzola et al., 1992; Chin-A-Woeng et al., 2003), contributes to biofilm formation *via* extracellular matrix production (Maddula et al., 2006; Maddula et al., 2008; Das et al., 2015; Wang et al., 2016), and may alter the availability of metals or other nutrients (Wang et al., 2011). Phenazines are redox active metabolites that have been studied extensively due largely to their capacity to generate host-damaging ROS (Mavrodi et al., 2001; Price-Whelan et al., 2006). Studies have shown that pseudomonads having genes required for phenazine production are prevalent in the rhizospheres of wheat grown under dryland production in Washington (Mavrodi et al., 2012) or grown in soils from dryland production fields in Texas (Mahmoudi, 2017). Recently, Mahmoudi et al. (2019) demonstrated that bacterial phenazine production contributed to plant drought-stress tolerance using *P. chlororaphis* 30-84 and isogenic derivative strains of *Pseudomonas chlororaphis* 30-84 deficient or enhanced in phenazine production compared to the wild type (Yu et al., 2018a). In that study, production of phenazines by *P. chlororaphis* 30-84 promoted drought-stress tolerance and resilience to repeated cycles of water deficit in seedlings of the winter wheat variety TAM 112. This was due in part to enhanced root system development that contributed to seedling survival and the drought-stress tolerance phenotype. Additionally, seedlings treated with phenazine-producing strains, especially the enhanced producer, managed osmotic stress better during the drought (as evident from shoot relative water content) and had less leaf tissue mortality and greater recovery after the drought than seedlings treated with no-inoculum controls. Recently, we showed that bacterial phenazine production in the rhizosphere contributed to the management of drought associated redox stress in the leaves (in prep). This led us to hypothesize that bacterial phenazine production may induce systemic tolerance of drought and other abiotic stresses such as salt stress that require osmotic and ROS stress management. Currently nothing is known regarding whether microbial phenazine production contributes to salt stress management.

In the present study, we explored this hypothesis using the same isogenic derivative strains of *Pseudomonas chlororaphis* 30-84 deficient or enhanced in phenazine production from the previous drought study. As in the previous study, the focus was on bacterially mediated improvements in stress tolerance during seedling establishment because this is often the most vulnerable stage and may greatly impact crop stand and yield (Liao et al., 2006). In addition to plant productivity under salt stress, we measured relative water content, and ROS accumulation and antioxidant enzyme activity in leaves following salt stress as indicators of osmotic stress tolerance and redox homeostasis. The use of phenazine deficient and overproducing strains enabled us to investigate whether microbial phenazine production in the rhizosphere was associated with improvements in any of these plant stress tolerance responses. We included TAM 112 (used in the previous drought study) and three additional commercial winter wheat varieties selected and widely used for dryland production in Texas, where phenazine-producing bacteria may be isolated. Because little is known regarding the salt tolerance of these wheat varieties, we used border cell production as a preliminary screen to select varieties that potentially varied in salt stress tolerance.

## Methods

### Bacterial Strains and Culture Conditions

Bacterial strains used in this study are shown in **Table 1**. A spontaneous rifampicin-resistant derivative of *Pseudomonas chlororaphis* subsp. *aureofaciens* 30-84 was used and is hereafter referred to as wild type (30-84 WT). A derivative of 30-84 WT with enhanced phenazine production (30-84 Enh) was obtained by removing a 90-bp sequence in the 5’ end untranslated region (5′-UTR) of *phzX*, the first gene in the phenazine biosynthetic operon, as described previously (Yu et al., 2018a). A phenazine deficient mutant (30-84 ZN) (Wood et al., 1997), was employed as a phenazine-deficient control. 30-84 WT and its derivatives were grown in Luria-Bertani medium (LB) medium containing 5 g of NaCl per liter, pH 7 (Lennox, 1955) at 28°C, with rapid agitation (200 rpm). Antibiotics were used where appropriate at the following concentrations: gentamicin (Gm) at 50 μg/ml and rifampicin (Rif) at 100 μg/ml.

**Table 1:** Bacterial strains used in this study.

Strain	Description^a^	Reference or source
***P. chlororaphis***		
30-84 WT	PCA^+^, 2-OH-PCA^+^, 2-OH-PHZ^+^, Rif^R^, Wild type	
30-84 Enh	PCA^+^, 2-OH-PCA^+^, 2-OH-PHZ^+^, Rif^R^, 90bp deletion at the 5’UTR of *phzX*	Yu et al., 2018b
30-84 ZN	Phz^-^, *phzB*::*lacZ*, Rif^R^	Wood et al., 1997

^a^Rif^R^ = rifampin resistance.

### Root Border Cell Production

Seeds of 20 winter wheat varieties were provided by Xuejin Dong, Texas A&M AgriLife Research, Uvalde, Texas and included 10 commercial varieties from the Oklahoma State University and Texas A&M University breeding programs and 10 proprietary commercial varieties recommended and widely used for dryland production in the Texas High Plains and Texas Rolling Plains. These varieties vary in development and their drought tolerance and disease and insect resistance (**Supplementary Table 1**).

Wheats seeds of each variety were surface sterilized and germinated within germination paper (10 seeds/paper) and maintained in the dark (28°C) for two days, as described previously (Mahmoudi et al., 2019). Uniformly sized seedlings were selected for border cells counts. The root tips from the primary root and two seminal roots were excised and incubated separately in 100 µl sterilized water with gently shaking for 10 min to release border cell from root. The border cells were collected, transferred to a hemocytometer, and counted using light microscopy (100 X). Data are reported as the average for the three root tips from five seedlings per wheat variety across three independent experiments.

### Bacterial Growth and Phenazine Production Under Salt Stress Treatment

Inoculum of each of strain was prepared by growing them separately overnight in LB broth. Cells were collected *via* centrifugation (after being washed with sterile water) and resuspended in LB, and then cell densities were adjusted to a standard optical density (OD_620_ = 1.0). The cultures were used to inoculate (1:50 ratio) the nonstressed control (no additional salt) or saline growth medium. Saline conditions were generated by the addition of NaCl (0%, 0.5%, 1.25%, 2.5%, 5%, and 10%, w/v, e.g., 85, 210, 425, 850, and 1,700 mM NaCl) to LB medium. This concentration gradient produced saline conditions similar to or exceeding (by ~10 X) the concentration measured in the saturation extracts from soil classified as saline to severely saline (~40–160 mM). At 12 and 24 h bacteria populations were quantified by spectroscopy (OD_620_) and then phenazines were extracted and quantified by spectroscopy (OD_367_, standardized to cell density) as described previously (Maddula et al., 2008; Wang et al., 2016). Briefly, cell cultures were acidified and phenazines were extracted in benzene, benzene was removed *via* evaporation, and phenazines were resuspended on 0.1 N NaOH.

### Effect of Bacterial Inoculation on Wheat Seed Germination Under Salt Stress Treatment

The experiment consisted of 2 × 2 factorial design: seeds of each variety treated with 30-84 WT or receiving no inoculum × exposure to the salt stress or no salt stress treatments. Inoculum of 30-84 WT was prepared as above. After 6 h, cells were pelleted, washed, and then cell densities were standardized to OD_620_ = 0.8 (≈ 10^8^ colony forming units/cfu) in 0.5% methylcellulose solution.

Wheats seeds of each variety were surface sterilized as described previously (Mahmoudi et al., 2019). Surface-sterilized seeds were submerged either in bacterial inoculum (room temperature, 10 min) or 0.5% methylcellulose solution (no-inoculum control) and then air dried (~3 h). For each treatment, 10 air dried seed were spaced apart on petri dishes on germination paper (three replicate plates/treatment). Seeds receiving a salt treatment were watered with 200mM NaCl or 120mM NaCl (5 ml), whereas nonsalt stressed seeds were treated with the same volume of sterile distilled water. These concentrations were selected because they bracket the concentration measured in the saturation extracts from soil classified as severely saline (160 mM). Three replicate petri dishes/treatment were placed in the dark at 28°C and germination percentage was measured after 3 days.

### Effect of Bacterial Inoculation on Wheat Seedling Growth Under Salt Stress

Four wheat varieties were selected for further testing: Iba, commercial variety CV 1, TAM 112 and TAM 113. For each variety, a 4 × 2 experimental design was used: seeds treated with either 30-84 WT, the phenazine-deficient mutant 30-84 ZN, or the phenazine-overproducing derivative 30-84 Enh, or no inoculum x salt stress or no stress treatments.

Wheats seeds of each variety were surface sterilized as above, placed onto germination paper, and maintained in the dark (28°C) for two days. Germinated seedlings that were uniform in growth were selected and treated with bacterial inoculum or methylcellulose (no inoculum control) as described above. Seedlings were grown separately in plastic tubes (Ray Leach Cone-tainers, 2.5-cm diameter × 16.5-cm long) containing steam sterilized (two times at 121°C for 1 h with a 24-h pause) planting medium (MetroMix 366, Sungro, Agawam, MA). Plants were grown for 7 days under well-watered (no salt) conditions (25°C, 16:8 light dark cycle, 150–170 μE^-1^ m^-1^ s^-1^, water twice weekly with 10 ml distilled water). After 7 days, the seedlings received either a salt stress treatment (10 ml of 200 mM NaCl, twice weekly) or no salt (10 ml distilled water). This concentration was selected because it is slightly above the concentration measured in the saturation extracts from soil classified severely saline (160 mM). After 3 weeks, seedlings were harvested, and roots were washed to remove adhering planting medium. Bacterial population sizes were determined from a random sample of replicates *via* serial dilution plating on LB agar supplemented with rifampicin. Shoot and root fresh weight (FW), turgid weight (TW, saturated leaf weight after 24 h), and dry weight (DW, 12 h at 65° C) were measured and shoot measurements were used to calculate relative water content as described previously (Mahmoudi et al., 2019). Productivity indices were calculated by standardizing shoot or root DW biomass to the DW shoot or root biomass of the no salt control, respectively. Data are from three independent experiments with five replicates seedlings/treatment.

### Effect of Bacterial Inoculation on ROS Accumulation and Antioxidant Enzyme Activity in Leaves

Hydrogen peroxide (H_2_O_2_) detection was performed *in situ* using a 3,3′-diaminobenzidine (DAB) staining method as described previously (Daudi and O’Brien, 2012). For all inoculation x stress treatments, three fully expanded leaves (third leaves) from separate wheat plants were obtained 2-week post salt stress treatment. Leaves were immediately vacuum-infiltrated with DAB (Sigma) staining solution (1 mg/ml DAB, 10mM Na_2_HPO_4_ and 0.05% Tween-20, pH 7.4) and incubated in the dark (3 h, gentle agitation). Leaves were fixed and chlorophyll destained using several washes with a 1:3:1 mixture of lactic acid:ethanol:glycerol (65°C, 6 h), mounted in Tris/glycerol, and examined under a dissecting microscope (1.5X) for the presence of reddish-brown precipitate.

Antioxidant enzyme (catalase) activity was measured in three fully expanded leaves taken from separate wheat plants 1-week post salt stress treatment based on previous methods (Kar and Mishra, 1976). Immediately after harvested, leaves were stored at –80°C. Samples were ground in liquid N_2_; 0.5 g of the grounded sample was homogenized in 1 ml PBS (pH 7.4) solution with protease inhibitor. The catalase (CAT) activity of the samples was quantified using spectrophotometry (NanoVue Plus, GE Healthcare, Piscata, NJ) to measure the decomposition of H_2_O_2_ over 10 min (as the decrease in absorbance at 240 nm). Data are from three independent experiments with three replicates/treatment (i.e., leaves taken from separate plants).

### Statistical Analyses

For ROS staining and catalase activity, measurements were made on three leaves from separate plants/treatment/experiment and the experiment was replicated independently three times. Data were analyzed by variety using a two-way ANOVA and Tukey’s test for multiple comparisons (P < 0.05, N=9). For border cell counts, data are reported as the average for the three root tips from five seedlings per wheat variety across three independent experiments. For germination rates, data are the average and standard error of the percentage of 10 seeds that germinated in three replicates. Root and shoot productivity and relative water content measurements were made on five seedlings/treatment/experiment and experiments were replicated three times. Productivity data were analyzed by variety using a one-way ANOVA and Dunnett’s test for comparison to the control (P < 0.05, N=15) using Real Statistics in Excel (http://www.real-statistics.com). Relative water content was analyzed using ANOVA as above. For bacterial growth and phenazine production, there were three replicates/treatment and three independent experiments were performed, and data were analyzed using ANOVA as above. Data were examined graphically to ensure they met the assumptions of the ANOVA.

## Results

### Root Border Cell Production

In the absence of any varietal information on salt stress tolerance, we prescreened 20 winter wheat varieties for potential salt sensitivity based on root border cell production, given the potential for mucilage secreted by border cells to contribute to salt tolerance. The lowest border cell producing varieties (~2,000/root tips) were TAM 304, Iba, and a commercial variety (CV 1) (**Supplementary Table 1**; **Supplementary Figure 1**). The highest border cell producing varieties were the drought tolerant varieties TAM 112 and Duster (~5,000/root tips). In an effort to obtain a spectrum of potential susceptibilities to salinity among varieties, based on these observations varieties Iba and CV 1 were included in the subsequent experiments as potentially salt sensitive varieties. TAM 112 used in a previous drought study (Mahmoudi et al., 2019) and TAM 113 (also a high border cell producer) were included as potentially more salt/stress tolerant varieties.

### Effect of Bacterial Inoculation on Wheat Seedling Growth Under Salt Stress

The effects of bacterial seed-inoculation on growth was measured by comparing seedlings of Iba, CV 1, TAM 112 and TAM 113 receiving no inoculum or inoculum comprised of 30-84 ZN, 30-84 WT, or 30-84 Enh grown in salt or no salt treatments. Prior to the application of salt stress treatment, all seedlings were grown 7 days without salt treatment and several replicate plants were sacrificed for bacterial population measurements *via* serial dilution. Bacterial populations of all three strains on all four varieties achieved populations greater than or equal to 10^7^ cfu/g dry weight root.

The wheat varieties varied in their response to the salt stress treatment in terms of cumulative above and below ground productivity and relative water content (RWC) of the above ground tissue (**Table 2**; **Supplementary Figure 2**). For example, compared to the no salt stress/no inoculum control, shoot productivity and root productivity of all salt-stressed/noninoculated varieties were reduced to 45%–63% (shoot) and 28%–44% (root) for varieties Iba, CV 1, and TAM 112. However, TAM 113 maintained shoot and root productivity at a level that was not significantly different from the no salt control. Salinity caused osmotic stress in all four varieties as evident from the drop in RWC compared to the nonstressed control plants, however in TAM 112 and TAM 113 the reduction was less (RWC = 66%) than observed for IBA and CV 1 (RWC < 40%).

**Table 2:** Productivity and Relative Water Content of seedlings grown under no salt or salt stress conditions treated with or without bacterial inoculum.

Variety Control/Stress Treatment	Inoculation Treatment*	PercentProductivity Shoot(P < 0.05)**	PercentProductivity Root(P < 0.05)	Relative Water Content***
**Iba**				
No Salt	**No Inoculum**	**100**	**100**	89 ± 4 a
	30-84 ZN	93	107	94 ± 1 a
	30-84 WT	124	108	90 ± 2 a
	30-84 Enh	157 **+**	114	91 ± 3 a
Salt	No Inoculum	48 **-**	28 **-**	38 ± 7 c
	30-84 ZN	79	28 **-**	67 ± 5 b
	30-84 WT	79	32 **-**	63 ± 9 b
	30-84 Enh	100	38 **-**	73 ± 5 b
**CV 1**				
No Salt	**No Inoculum**	**100**	**100**	92 ± 4 a
	30-84 ZN	118	99	88 ± 1 a
	30-84 WT	166 **+**	107	88 ± 2 a
	30-84 Enh	170 **+**	111	91 ± 3 a
Salt	No Inoculum	63 **-**	30 **-**	36 ± 7 c
	30-84 ZN	89	29 **-**	63 ± 5 b
	30-84 WT	96	34 **-**	70 ± 9 b
	30-84 Enh	115	47 **-**	79 ± 5 b
**TAM 112**				
No Salt	**No Inoculum**	**100**	**100**	92 ± 11 a
	30-84 ZN	102	107	91 ± 8 a
	30-84 WT	100	107	99 ± 8 a
	30-84 Enh	122	130 **+**	93 ± 8 a
Salt	No Inoculum	45 **-**	44 **-**	66 ± 7 b
	30-84 ZN	67 **-**	44 **-**	66 ± 3 b
	30-84 WT	71	56 **-**	72 ± 8 b
	30-84 Enh	92	56 **-**	79 ± 13 ab
**TAM 113**				
No Salt	**No Inoculum**	**100**	**100**	92 ± 3 a
	30-84 ZN	102	139 **+**	99 ± 2 a
	30-84 WT	106	138 **+**	98 ± 4 a
	30-84 Enh	108	138 **+**	94 ± 5 a
Salt	No Inoculum	93	66 ± 9 b
	30-84 ZN	81	82	76 ± 5 b
	30-84 WT	100	125	77 ± 9 b
	30-84 Enh	104	115	82 ± 6 b

*Three-day old seedlings received either no inoculum (methylcellulose control) or 30-84 ZN, 30-84 WT, or 30-84 Enh inoculum and were grown for one week without stress. Plants then received no salt stress (control) or a salt stress treatment (200 mM NaCl) for 3 weeks and dry weight biomass of shoots and roots were measured. Data are from three independent experiments with five replicates seedlings/variety/treatment.

** Productivity is expressed as the shoot or root dry weight biomass standardized to the shoot or root dry weight biomass of the no salt/no inoculum control (bold), respectively. Productivity measurements were analyzed by variety using a one-way ANOVA and Dunnett’s test for comparison to the control (P < 0.05, N=15). + and – indicate whether the productivity is significantly greater or less than the control, respectively.

*** Means and standard deviations of the relative water content of the above ground tissue were compared by variety using a two-way ANOVA and Tukey’s test for multiple comparisons (P < 0.05, N=3) and letters indicate significant differences.

In the no salt stress condition, seed treatment with 30-84 Enh promoted above ground growth of Iba and CV 1 and below ground growth of TAM 112 and TAM 113. However, the growth promoting effect on TAM 113 was not dependent on phenazine production as 30-84ZN also promoted plant growth. Under salt-stressed conditions, seed treatment with 30-84 WT, 30-84 Enh or 30-84ZN enabled seedlings of most varieties to maintain shoot productivity equivalent to the no stress/no inoculum control, however for TAM 112 growth promotion occurred only when plants were treated with phenazine producing bacteria. Under the salt stress condition, seed treatment with 30-84 WT, 30-84 Enh or 30-84 ZN enabled TAM 113 to maintain root productivity. These data indicate that seed treatment with *P. chlororaphis* 30-84 plays a role in promoting growth and helping seedlings tolerate salt stress, but the requirement for phenazine production depends on wheat variety.

### Effect of Bacterial Inoculation on ROS Accumulation and Antioxidant Enzyme Activity in Leaves

**Figure 1:**
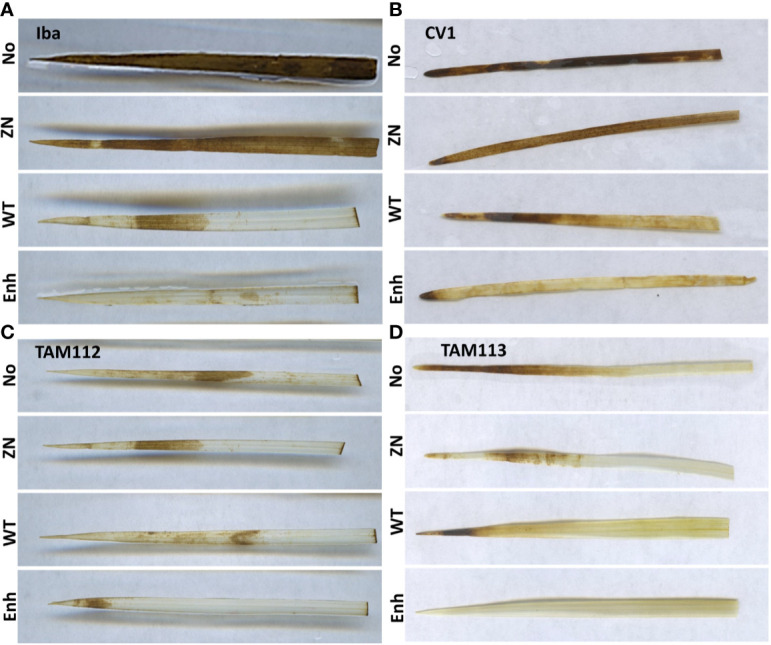
The effect of no inoculum or inoculation with 30-84 ZN, 30-84 WT, or 30-84 Enh (top to bottom, NO, ZN, WT, Enh, respectively) on reactive oxygen species (ROS) accumulation in leaves obtained from seedlings of different wheat varieties grown under the salt stress condition. **(A)** Iba, **(B)** CV 1, **(C)** TAM 112, and **(D)** TAM 113. Seeds were treated with either no inoculum (methylcellulose) or 30-84 ZN, 30-84 WT or 30-84 Enh and grown one week without stress. Then plants were exposed to salt stress (200mM NaCl). DAB staining revealed ROS accumulation in fully expanded leaves (third leaves) from three separate wheat plants/variety/experiment in three separate experiments. Representative images are shown.

The effect of seed inoculation with 30-84 ZN, 30-84 WT, or 30-84 Enh on ROS (H_2_O_2_) accumulation in seedling leaves was measured using three fully expanded leaves (third leaves) from separate wheat plants collected two-weeks post salt stress treatment (**Figure 1**). Under the no salt condition, almost no ROS accumulation was observed in the leaves obtained from any of the four wheat varieties and all seed-inoculation treatments (data not shown). For seedlings grown in the salt treatment, the leaves of CV 1 and Iba obtained from noninoculated seedlings or seedlings seed-inoculated with 30-84 ZN displayed high levels of ROS stress, whereas TAM 112 and TAM 113 leaf ROS accumulation was lower. In all varieties, seed-inoculation with 30-84 WT and especially 30-84 Enh resulted in little ROS accumulation in the leaves of seedlings when grown in the salt treatment. These observations indicated that seed-inoculation of wheat with 30-84 WT or 30-84 Enh reduced ROS accumulation as compared to noninoculated seedlings, in the salt stress treatment.

**Figure 2:**
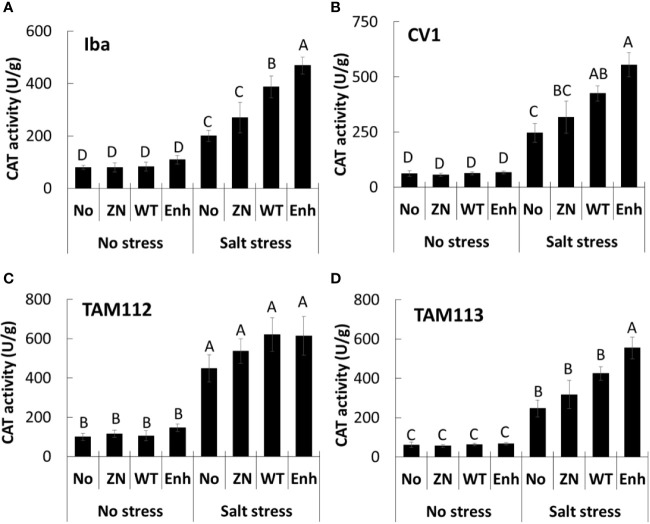
The effect of no inoculum or inoculation with 30-84 ZN, 30-84 WT, or 30-84 Enh on catalase enzyme activity in leaves obtained from seedlings of different wheat varieties in no salt and salt stress treatments. **(A)** Iba, **(B)** CV 1, **(C)** TAM 112, and **(D)** TAM 113. Seeds were treated with either no inoculum (methylcellulose) or 30-84 ZN, 30-84 WT, or 30-84 Enh and grown one week without stress and the received either a salt stress (200mM NaCl) or no salt stress. Catalase activity was measured in fully expanded leaves (third leaves) from three different wheat plants/variety/experiment in three separate experiments. Data are the means and standard errors. Data were analyzed by variety using a two-way ANOVA and Tukey’s test for multiple comparisons (P < 0.05, N=9) and letters indicate significant differences.

Catalase activity was significantly greater in seedlings grown in the salt stress treatment compared to the nonstressed treatment (200–475 U/g versus ≤ 150 U/g, respectively, **Figure 2**). Moreover, in the salt stress treatment catalase activity was significantly higher in Iba and CV 1 leaves from plants seed-inoculated with 30-84 WT or 30-84 Enh (400–500 U/g) compared to the noninoculated control, whereas for TAM 112 and TAM 113 differences were less obvious. These observations suggest that seed-inoculation of plants with 30-84 WT or 30-84 Enh enhanced catalase enzyme activity in the leaves of wheat seedlings grown in the salt stress condition, consistent with the reduction in leaf ROS accumulation we observed.

### Effect of Bacterial Inoculation on Wheat Seed Germination Under Salt Stress Treatment

Percent germination rates of seeds of 20 winter wheat varieties with and without bacterial inoculum and with and without salt treatment were compared. Seeds from the different wheat varieties were either treated with 30-84 WT or received no inoculum (methylcellulose control) and either watered with distilled water or solutions containing 200 mM or 120 mM NaCl solution. After 3 days, average germination rates of all 20 varieties in the no salt stress treatment, whether receiving no inoculum or 30-84 WT inoculum were 95% ± 1% and 96% ± 1%, respectively. No seeds germinated from the 200 mM treatment. For the 120 mM treatment, average germination rates of all wheat varieties were markedly reduced and ranged from 33%–70% with or without bacterial inoculum (**Supplementary Table 1**). Several of the varieties including Iba and CV 1, and TAM 304 had germination rates in the 30-45% range under saline conditions (e.g., 37% ± 3%, 43% ± 3%, 33% ± 8%, respectively), however inoculation of seeds with 30-84 WT improved germination of both Iba and CV 1, but not TAM 304 (53% ± 8%, 63% ± 8%, and 47% ± 8%, respectively). At the high end of the spectrum, under saline conditions the germination percentages of the widely used drought tolerant TAM 112, TAM 113, and Duster were 63% ± 3%, 47% ± 8%, and 70% ± 6%, respectively and were not improved by inoculation with 30-84 WT (63% ± 8%, 40% ± 5%, and 70% ± 6%), indicating seed inoculation with 30-84 WT mediated saline inhibition of germination in some, but not all varieties.

### Bacterial Growth and Phenazine Production Under Salt Stress Treatment

**Figure 3:**
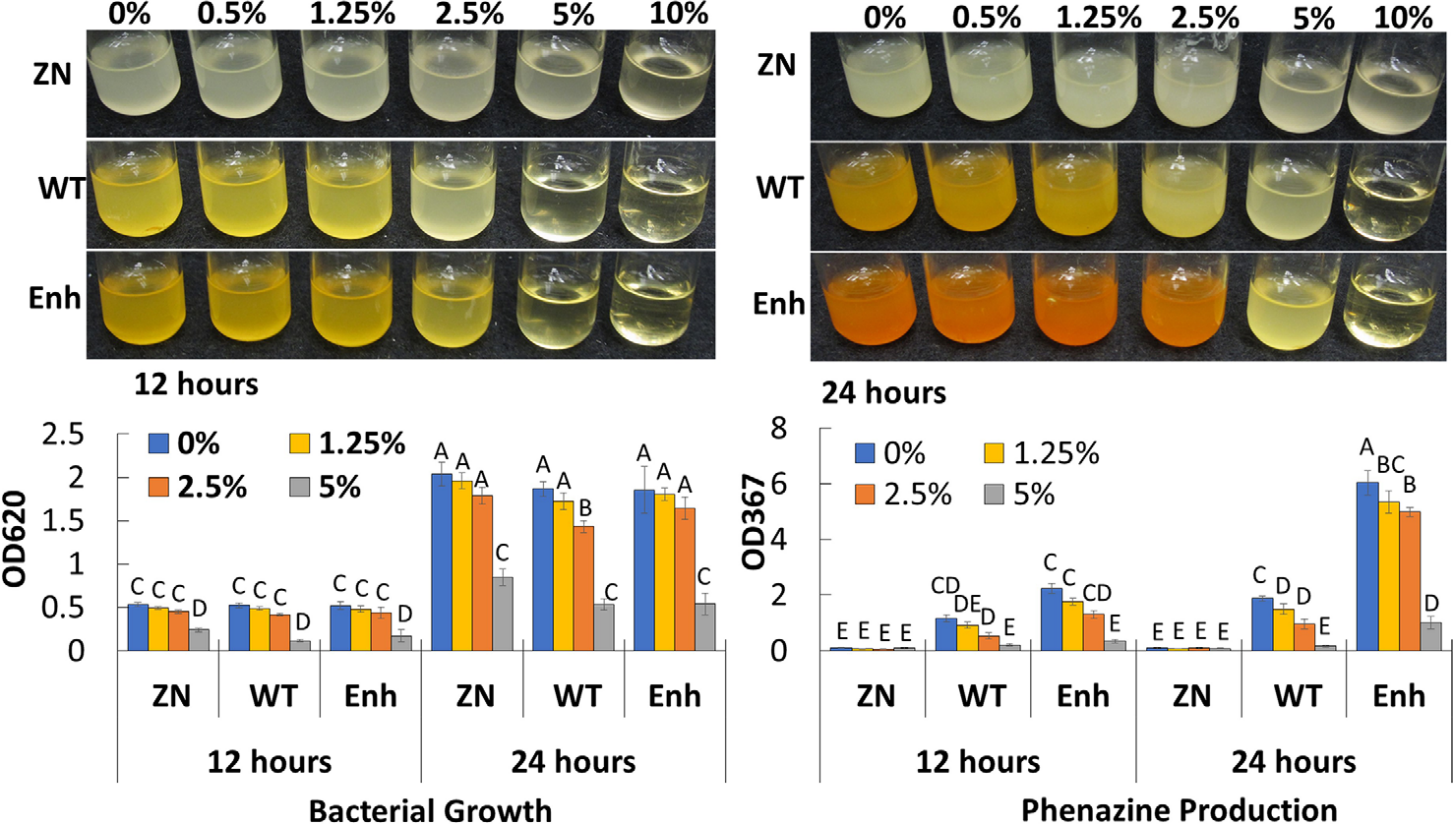
Growth and phenazine production of *P. chlororaphis* 30-84 WT and derivatives under increasing salt concentrations. Bacterial populations and phenazine production for 30-84WT, 30-84ZN, and 30-84 Enh were measured after 12 and 24 h in Luria-Bertani medium (LB) amended with different salt concentrations (0%, 0.5%, 1.25%, 2.5%, 5%, and 10% NaCl, w/v, e.g., 85, 210, 425, 850, and 1,700 mM NaCl). Bacterial populations and phenazine production were quantified spectrophotometrically (OD_620_ and OD_367_, respectively) and means and standard errors are shown. Data were analyzed using a two-way ANOVA and Tukey’s test for multiple comparisons (P < 0.05, N=3) and letters indicate significant differences.

To determine the extent to which salt concentration affects bacterial growth and phenazine production, 30-84 WT and derivatives deficient (30-84 ZN) or enhanced (30-84 Enh) in phenazine production were grown in LB media amended with no NaCl or different concentrations of NaCl (0.5%, 1.25%, 2.5%, 5%, and 10%, w/v, e.g. 85–1,700 mM), and bacterial populations and phenazine production was quantified at 12 and 24 h (**Figure 3**). Because phenazine production is regulated *via* quorum sensing in a cell density-dependent manner, it was important to consider the effects of salt on population density. Cell densities of 30-84 WT and 30-84 Enh when grown in the lowest salt concentrations (0.5 to 2.5%, e.g. ≤ 425 mM) were only slightly different from the no-salt treatment, however phenazine production by 30-84 WT was reduced at 2.5%, but not at 1.25% (~210 mM). Compared to the no salt control, at the 5% salt concentration (850 mM) bacterial growth and phenazine production were significantly reduced. None of the derivatives grew in 10% NaCl. These data suggest that when *P. chlororaphis* 30-84 is grown in saline conditions similar to those measured in the saturation extracts from soil classified as saline to severely saline (~40–160 mM), bacterial growth and phenazine production are not substantially impaired.

## Discussion

This study demonstrated that the four wheat varieties examined responded differently to the salt stress treatment and the benefits derived from colonization by *P. chlororaphis* 30-84. In all varieties, the salt stress treatment reduced seed germination rates. In seedlings of the four varieties tested, salt stress reduced shoot water content, increased reactive oxygen species levels in leaves, and in three of four varieties, reduced shoot and root production compared to the no salt stress treatment. It was interesting that TAM 113 seedlings were able to maintain biomass production in the salt treatment despite experiencing osmotic stress (RWC=66%), indicating seedlings of this variety may be less susceptible to growth inhibition at this level of salinity. Also, root border cell production turned out to be a somewhat good predictor of salinity tolerance, although our sample size was small, and more validation is required. Iba and CV 1, which produce 60% fewer border cells than TAM 112, experienced greater leaf redox stress, significant reductions in relative water content under saline conditions, and reduced germination rates under saline conditions. They also gained significant improvements in these traits with bacterial inoculation. Previous work showed that in responses to metal ions such as aluminum, copper, lead, cadmium, mercury, arsenic, and iron mucilage from border cells of various crops including cotton, cereals, and legumes expands to trap metal ions in a concentration‐dependent manner and when border cells are removed, plants experience greater heavy metal toxicity (reviewed in Hawes et al., 2016). Although not specifically addressed in this study, potential roles for border cells in helping plants protect themselves from salt ions and improving plant responses to the activities of stress-mediating microbes deserves further consideration.

The most striking finding of our study was the association between bacterial phenazine production in the rhizosphere and enhanced catalase activity and redox stress management in the leaves in all four varieties. *P. chlororaphis* 30-84 produces several phenazines, but only two in significant abundance: phenazine-1-carboxylic acid (PCA) and 2-hydroxy-PCA (2-OH-PCA). It is well established that salt stress causes the accumulation ROS such as O_2_·^-^, H_2_O_2_, and ·OH in leaves, leading to cytoplasmic and nuclear membrane peroxidation (Zhu, 2016; Yuan et al., 2017; Yuan et al., 2018c). Moreover, cellular redox state serves as a sensor of environmental changes that impose oxidative stress, and plants have efficient ROS scavenging systems to avoid ROS accumulation to maintain cellular homeostasis (Zhu, 2016). Previous studies showed that under saline conditions PGPR may augment plant ROS scavenging systems *via* transcriptional induction of genes encoding plant antioxidant enzymes such as ascorbate peroxidase (APX), superoxide dismutase (SOD), peroxidase (POD), catalase (CAT), and glutathione peroxidase (GPX) (Bharti et al., 2016). In the present study under saline conditions, microscopic observation of DAB-staining in leaves revealed that ROS accumulation was located on the cell membranes and in apoplasts of leaf cells (**Figure 1**). However, inoculation of seedlings with phenazine-producing *P. chlororaphis* 30-84 visibly diminished H_2_O_2_ accumulation in all varieties and significantly promoted catalase activity in leaves of Iba and CV 1, especially plants treated with 30-84 Enh as compared to plants treated with 30-84 ZN or receiving no inoculum. Recently using the same staining protocol, we found that seed inoculation of TAM 112 with 30-84 WT or 30-84 Enh, but not 30-84 ZN, also visibly diminished H_2_O_2_ accumulation in the leaves of drought stressed seedlings. These observations suggest that bacterial phenazine production promotes tolerance of drought and salt stress in wheat seedlings in part through the modulation of ROS-mediated signaling pathways, although the precise mechanism needs further study. As indicated from previous work showing the broad transcriptomic consequences associated with the lack or overexpression of phenazines (Wang et al., 2016), it is reasonable that the microbial mechanisms underlying these results may be more complex and not solely due to the phenazines.

Previous studies showed that in addition to suppression of seedling disease, PGPR contribute to growth promotion and salt stress tolerance directly and indirectly *via* the production of bioactive compounds and modifications to the rhizosphere environment *via* extensive biofilm formation, respectively (Yang et al., 2009). For example, PGPR are known to enhance germination rates, improve nutrient status, and alleviate salt stress in wheat seedlings *via* the production of: indole acetic acid (IAA), which mediates lateral branching, resulting in increased fine root length, surface area, tip number, and water and primary nutrient uptake (Ramadoss et al., 2013; Egamberdieva et al., 2015); the enzyme 1-aminocyclopropane-1-carboxylate (ACC)-deaminase, involved in the degradation of the ethylene precursor ACC, resulting in improved root growth and stress tolerance (Bal et al., 2013); antioxidant enzymes such as catalase involved in ROS degradation and management of redox stress (Jha and Subramanian, 2014); siderophores and phosphate solubilizing compounds and other undetermined traits important for improved nutritional status (Nadeem et al., 2014; Rajkumar et al., 2017); and EPS matrix, which helps improve soil structure, increase soil water retention, and reduce the available Na^+^ (Naseem and Bano, 2014; Banerjee et al., 2019). *P. chlororaphis* 30-84 (including 30-84ZN) produces some of these bioactive compounds, including IAA, ACC-deaminase, pyoverdine class siderophores, and antioxidants (Mahmoudi et al., 2019). It is likely these contributed to the effects of both phenazine-producing and nonproducing strains on growth promotion and osmotic tolerance in seedlings. An interesting question is whether the production of these bioactive compounds is correlated with phenazine production, thus contributing to the beneficial effects associated with phenazine production. Previous research showed that IAA production by 30-84ZN was higher than by 30-84WT in static floating biofilms, whereas the expression of genes encoding ACC-deaminase were similar in both strains, and the genes encoding pyoverdine biosynthesis and catalase and other enzymes involved in redox stress management were expressed at higher levels in 30-84WT than 30-84ZN, although production of these enzymes or compounds were not measured (Wang et al., 2016). In addition to the production of bioactive compounds, *P. chlororaphis* 30-84 makes substantial EPS matrix, which could serve to buffer the plant to some extent from the saline environment. Consistent with other phenazine-producing strains, biofilm development is partially dependent on phenazine production (Wang et al., 2016; Letourneau et al., 2018).

Previous studies demonstrated inhibitory effects of NaCl on phenazine production as observed in this study (Girard and Rigali, 2011). Thus, we were interested in whether phenazines would be produced when the strains were exposed to the salinity conditions used in our plant assays. For *P. chlororaphis* 30-84, these effects were only observed at extremely high salt concentrations (425-850 mM) that are unlikely to occur in saline soil water. These observations suggest that it is unlikely that the production of phenazines was diminished in the saline treatment, but further testing is warranted.

In summary, the results of our study using phenazine deficient and overexpressing derivatives as seed-inoculants demonstrate that phenazine-producing rhizobacteria promote plant growth and reduce redox and osmotic stress in seedlings grown in saline conditions, and that the reduction in redox stress is associated with phenazine production. These findings indicate a critical starting point for investigating the mechanisms underlying the relationship between phenazine production by rhizosphere-colonizing bacteria and plant stress tolerance, and ongoing work is focused on how bacterial phenazine production alters plant signaling pathways involved in redox homeostasis. Results from the present study, together with those from previous studies (Yu et al., 2018b; Mahmoudi et al., 2019), demonstrate the potential for phenazine-producing strains prevalent in dryland wheat production areas to suppress plant diseases and promote drought and salt stress tolerance in wheat seedlings. These studies also suggest that breeding wheat for effective recruitment and response to native phenazine-producing bacteria could be a viable approach for improving wheat stress tolerance.

## Data Availability Statement

The datasets generated during this study are available on request to the corresponding author.

## Author Contributions

PY, LP, and EP designed the experiments. PY and EB carried out the experiments and generated original data, with technical advice from HP. PY and EP performed the data analysis. PY, EB, HP, LP, and EP contributed to the interpretation of results, and PY, HP, and EP contributed to the first draft of the manuscript. EP provided project supervision, and EP and HP provided major contribution to the final draft. All authors contributed to the article and approved the submitted version.

## Funding

This research was funded in part by support to EP from DOE-Office of Energy Efficiency and Renewable Energy Award DE-EE0007104 and the College of Agriculture and Life Sciences.

## Conflict of Interest

The authors declare that the research was conducted in the absence of any commercial or financial relationships that could be construed as a potential conflict of interest.
