# Supplementary material for: Phenazine-Producing Rhizobacteria Promote Plant Growth and Reduce Redox and Osmotic Stress in Wheat Seedlings Under Saline Conditions
Source: Front Plant Sci. 2020 Sep 29;11:575314. doi: 10.3389/fpls.2020.575314 (PMC7550623; doi:10.3389/fpls.2020.575314)
Supplement: Supplementary file 1 [file DataSheet_1.pdf]

Supplementary Figure 1

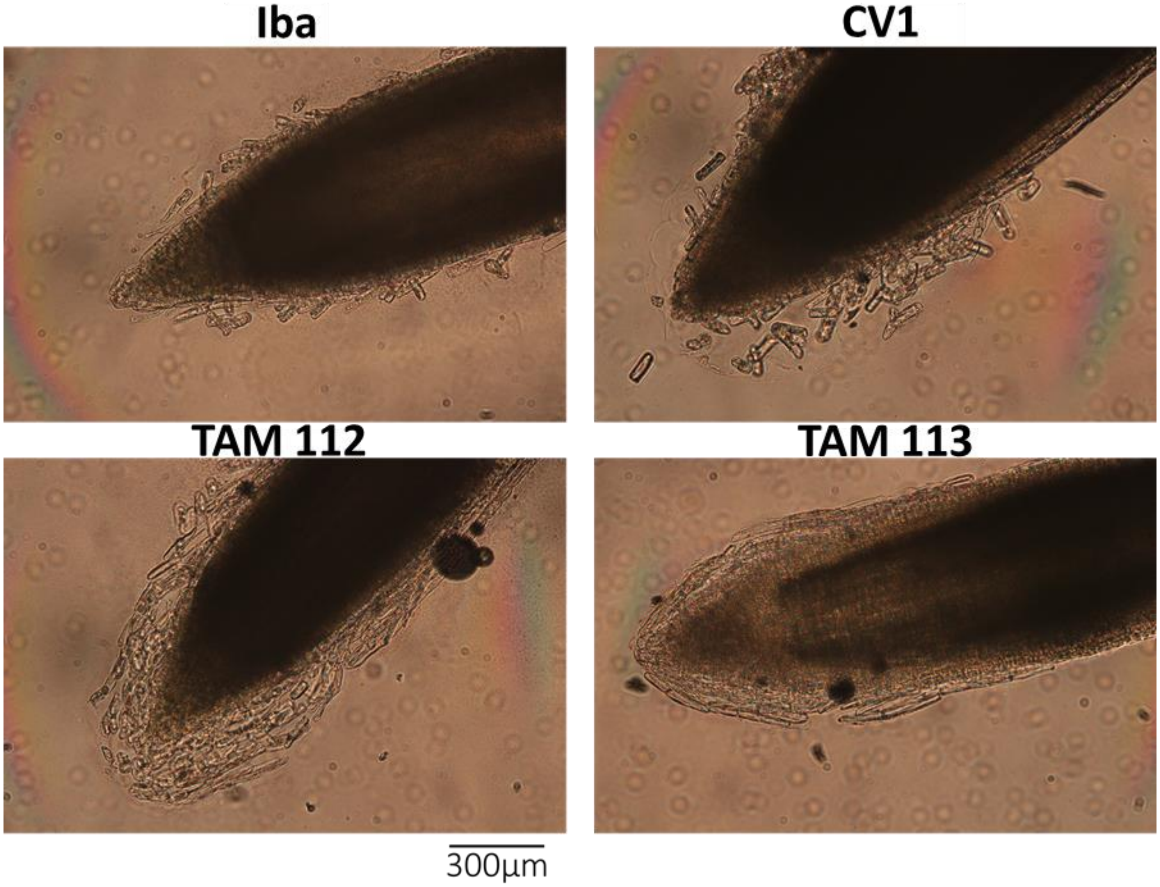

Supplementary Figure 1: Images of border cells adhering to the primary root of 2-day old seedlings of wheat varieties: Iba, CV 1, TAM 112 and TAM 113 obtained using a light microscope (100X). The images were obtained within 10-20 s of immersion in water, before border cell release. The scale bar indicates 300 µm.

Supplementary Figure 2

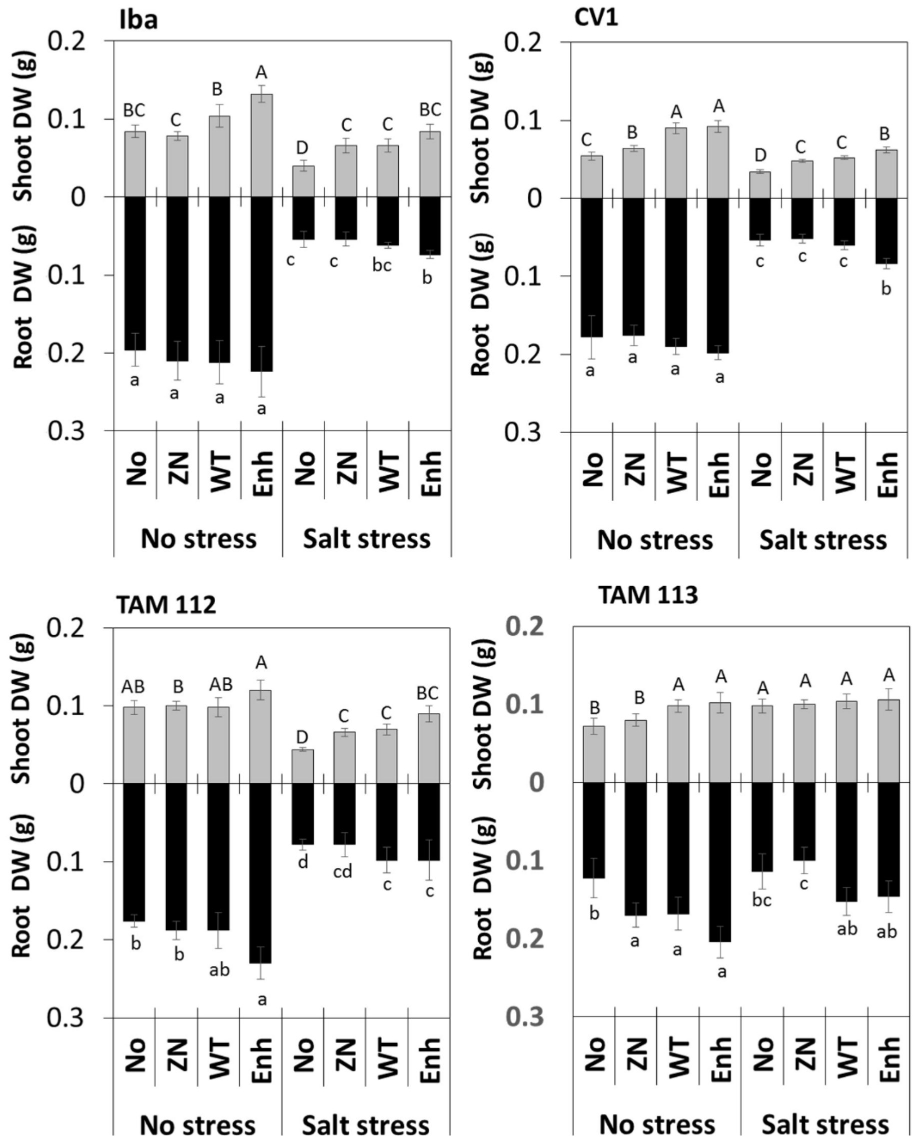

Supplementary Figure 2. The effect of no inoculum or inoculation with 30-84 ZN, 30-84 WT or 30-84 Enh on the growth of seedlings of different wheat varieties in no salt and salt stress treatments. A. Iba, B. CV 1, C. TAM 112, and D. TAM 113. Seeds were treated with either no inoculum (methylcellulose) or 30-84 ZN, 30-84 WT or 30-84 Enh and grown one week without stress and the received either a salt stress (200mM NaCl) or no salt stress. Shoot and Root Dry Weight production were measured, and data are the means and standard errors 5 replicate seedlings/variety/treatment. Data were analyzed by variety using a two-way ANOVA and Tukey's 17 test for multiple comparisons ($P < 0.05$, N=15) and letters indicate significant differences.

Supplementary Table 1. Number of border cells produced by 2 day-old seedlings and the germination rate of seeds treated with 12 mM NaCl, without (no inoculum) or with bacterial inoculum (30-84 WT).

Variety Name*	Reference	Border Cell Number **	Germination Rate***	
			No Inoculum	30-84 WT
**TAM 112**	Texas A&M University, *Journal of Plant Registrations*, **8**, 3, (291-297), (2014).	**5287 ± 763**	**63**	**63**
**TAM 113**	Texas A&M University, *Journal of Plant Registrations*, **7**, 1, (63-68), (2013).	**3597 ± 556**	**47**	**40**
**TAM 114**	Texas A&M University, *Journal of Plant Registrations*, **12**, 3, (367-372), (2018).	4333 ± 1155	50	47
**TAM 304**	Texas A&M University, *Journal of Plant Registrations*, **9**, 3, (331-337), (2015).	2037 ± 387	33	47
**TAM 305**	Texas A&M University, *Journal of Plant Registrations*, **9**, 3, 325-330), (2015).	3207 ± 571	47	40
**TAM 401**	Texas A&M University, *Journal of Plant Registrations*, **6**, 1, (60-65), (2012).	4507 ± 314	30	40
**Bentley**	Oklahoma State University, http://wheat.okstate.edu/variety-characteristics-1/variety-characteristics-1/varieties/Bentleybrochurepage2015.pdf	2990 ± 75	53	47
**Duster**	Oklahoma Agricultural Experiment Station and the USDA-ARS, Journal of Plant Registrations, Vol. 6, No. 1, January 2012, http://wheat.okstate.edu/variety-characteristics-1/variety-characteristics-1/varieties/duster-hrww	5330 ± 75	70	70
**Gallagher**	Oklahoma State University, http://wheat.okstate.edu/variety-characteristics-1/variety-characteristics-1/varieties/gallagher	3640 ± 645	50	50
**Iba**	Oklahoma State University , http://wheat.okstate.edu/variety-characteristics-1/variety-characteristics-1/varieties/iba	**2167 ± 340**	**37**	**53**
**CV 1[†]**	Proprietary	**2123 ± 576**	**43**	**63**
**CV 2**	Proprietary	3857 ± 615	37	43
**CV 3**	Proprietary	3943 ± 436	47	50
**CV 4**	Proprietary	3770 ± 544	37	37
**CV 5**	Proprietary	3943 ± 115	43	47

**CV 6**	Proprietary	3597 ± 243	50	50
**CV 7**	Proprietary	3943 ± 1046	47	43
**CV 8**	Proprietary	4333 ± 605	33	40
**CV 9**	Proprietary	4680 ± 544	47	53
**CV 10**	Proprietary	3727 ± 841	50	47

* In addition to the named varieties, 10 commercial varieties were used in this study and information is withheld for proprietary reasons.

** Mean border cell number (three root tips from 5 seedlings per wheat variety/experiment, 3 independent experiments) ± standard error.

*** Mean germination rates (10 seeds/plate, 3 replicate plates/treatment) under salt stress conditions (120 mM NaCl) for seeds pre-treated with no bacterial inoculum (methylcellulose as a control) or 30-84 WT inoculum.

† This proprietary variety has the following resistance ratings: Fusarium Head Blight (Scab) 6, Leaf Rust 4, Soil-Borne Mosaic 2, Tan Spot 7, Wheat Stem Sawfly 2, Wheat Streak Mosaic 5, Yellow (Stripe) Rust 4 (10 point scale). Information on traits for all other varieties can be found in the references provided.
